# Relative hyperlactatemia and hospital mortality in critically ill patients: a retrospective multi-centre study

**DOI:** 10.1186/cc8888

**Published:** 2010-02-24

**Authors:** Alistair D Nichol, Moritoki Egi, Ville Pettila, Rinaldo Bellomo, Craig French, Graeme Hart, Andrew Davies, Edward Stachowski, Michael C Reade, Michael Bailey, David James Cooper

**Affiliations:** 1 Australian and New Zealand Intensive Care-Research Centre, School of Public Health and Preventive Medicine, Monash University, Alfred Hospital Campus, 75 Commercial Road, Prahran, VIC 31821, Australia; 2 Department of Anaesthesiology and Resuscitology, 2-5-1 Shikata-cho, Okayama, 700-8558, Japan; 3 Department of Intensive Care, The Alfred Hospital, 75 Commercial Road, Prahran, VIC 31821, Australia; 4 Department of Intensive Care, The Austin Hospital, 145 Studley Road, Heidelberg, VIC 3084, Australia; 5 Department of Intensive Care, The Western Hospital, 148 Gordon Street, Footscray, VIC 3011, Australia; 6 Department of Intensive Care, Westmead Hospital, Darcy Road & Hawksebury Road, Westmead, Sydney, NSW 2145, Australia

## Abstract

**Introduction:**

Higher lactate concentrations within the normal reference range (*relative hyperlactatemia*) are not considered clinically significant. We tested the hypothesis that relative hyperlactatemia is independently associated with an increased risk of hospital death.

**Methods:**

This observational study examined a prospectively obtained intensive care database of 7,155 consecutive critically ill patients admitted to the Intensive Care Units (ICUs) of four Australian university hospitals. We assessed the relationship between ICU admission lactate, maximal lactate and time-weighted lactate levels and hospital outcome in all patients and also in those patients whose lactate concentrations (admission n = 3,964, maximal n = 2,511, and time-weighted n = 4,584) were under 2 mmol.L^-1 ^(i.e. relative hyperlactatemia).

**Results:**

We obtained 172,723 lactate measurements. Higher admission and time-weightedlactate concentration within the reference range was independently associated with increased hospital mortality (admission odds ratio (OR) 2.1, 95% confidence interval (CI) 1.3 to 3.5, *P *= 0.01; time-weighted OR 3.7, 95% CI 1.9 to 7.00, *P *< 0.0001). This significant association was first detectable at lactate concentrations > 0.75 mmol.L^-1^. Furthermore, in patients whose lactate ever exceeded 2 mmol.L^-1^, higher time-weighted lactate remained strongly associated with higher hospital mortality (OR 4.8, 95% CI 1.8 to 12.4, *P *< 0.001).

**Conclusions:**

In critically ill patients, relative hyperlactataemia is independently associated with increased hospital mortality. Blood lactate concentrations > 0.75 mmol.L^-1 ^can be used by clinicians to identify patients at higher risk of death. The current reference range for lactate in the critically ill may need to be re-assessed.

## Introduction

In healthy individuals there is a continuous cycle of lactate production and metabolism, which ensures that blood lactate concentrations are normally low [1,2]. Higher blood lactate concentrations occur when lactate production exceeds clearance, when clearance capacity is decreased or more frequently when both occur simultaneously [3,4]. Elevated blood lactate concentrations above the accepted *normal *reference range (absolute hyperlactataemia) are common and associated with increased hospital mortality in the critically ill [5-12]. Their usefulness in identifying critically ill patients at higher risk of death has led to the adoption of lactate measurement in most blood gas analyzers and the frequent measurement of lactate in the critically ill.

While the normal lactate concentration in unstressed individuals is 1.0 ± 0.5 mmol.L^-1 ^[1,2], patients with critical illness are considered to have normal lactate levels at concentrations of less than 2 mmol.L^-1 ^[13]. Furthermore, this 2 mmol.L^-1 ^cut off may be considered to be a conservative threshold as some have suggested that a level of up to 4 mmol.L^-1 ^is within the normal limits [14].

However, it is unknown whether a higher blood lactate concentration within the current reference range (relative hyperlactataemia) might also be associated with increased hospital mortality. This knowledge would be clinically important because the currently used upper reference limit for lactatemia may fail to identify many patients who are at higher risk of death.

We hypothesized that higher blood lactate concentrations within the reference range would be associated with an increased risk of hospital death and investigated the relationship between ICU admission, maximal and time-weighted blood lactate concentrations and hospital mortality in a large cohort of critically ill patients.

## Materials and methods

The data collection and the data analysis for this study are part of ongoing de-identified data auditing processes across the participating hospitals, which have all waived the need for informed consent. The Austin Hospital Ethics Committee approved the study.

### Study population and data sources

We conducted this study as a four-centre retrospective investigation of a prospectively gathered intensive care database. Four Australian university teaching hospital intensive care units enrolled patients in this study. We included all patients admitted to these ICUs from January 2000 to October 2004.

The blood lactate concentration data used for this study were stored and retrieved electronically. We obtained age, sex, use of mechanical ventilation, reason for ICU admission, surgical and non-surgical divided into (trauma, cardiac/vascular, gastrointestinal tract, neurological and thoracic/respiratory diseases), and Acute Physiology and Chronic Health Evaluation (APACHE) II score [15] from the electronic data repositories of each ICU, using prospectively collected data as part of a continuing data collection by the Australian and New Zealand Intensive Care Society - Centre for Outcome and Resources Evaluation (ANZICS-CORE). We coded admission diagnosis by APACHE III system used by the ANZICS-CORE - Adult Patient Database [16].

All patients had initial arterial lactate and blood gas measured by blood gas analyser (Rapilab, Bayer Australia, Sydney, NSW, Australia, upper normal limit 2.00 mmol.L^-1^) at the time of admission to the ICU. The timing of repeat measurements was at the discretion of the managing critical care team. All subsequent blood lactate measurements were performed using the same blood-gas analyzer in each hospital. A *normal *(within reference) lactate was defined as a concentration between 0.00 and 2.00 mmol.L^-1 ^[13]. Laboratories in the participating hospitals comply with standards of the National Association of Testing Authorities [17] and the Royal College of Pathologists of Australasia [18].

### Statistical Analysis

We used the ICU admission (Lac_ADM_) and maximal (Lac_MAX_) blood lactate concentrations to indicate the admission and highest value recorded while in the ICU. We first assessed blood lactate concentration in all patients and second, in those patients whose ICU admission (Lac_ADM_), and maximal (Lac_MAX_) blood lactate concentrations never exceeded the *normal *reference range (that is, < 2 mmol.L^-1^). In addition, to avoid the potential effect of surveillance bias due to the increased blood lactate monitoring in more severely ill patients, we calculated the time-weighted lactate concentration (Lac_TW_). This time-weighted method is more representative of the true lactate level during the ICU stay than the arithmetic mean, as it assumes a linear trend between each individual lactate measurement for each patient during their ICU stay. This method was modified from, and used in accordance with, an approach previously used by Finney et al to describe hyperglycaemia [19].

As the relationship between Lac_ADM_, Lac_MAX_, Lac_TW _and mortality was expected not to be linear in nature, categorical variables were created. We divided lactate into four bands: *normal *(0.00 to 2.00 mmol.L^-1^); mild hyperlactemia (2.01 to 4.00 mmol.L^-1^); moderate hyperlactatemia (4.01 to 6.00 mmol.L^-1^) and severe hyperlactatemia (> 6.01 mmol.L^-1^) for comparison.

The *normal *range of lactate (0.00 to 2.00 mmol.L^-1^) was subsequently divided into eight bands. However, due to the small number of patients with values under < 0.75 mmol.L^-1 ^we combined the three lower octiles to achieve adequate size for statistical comparison. We therefore compared: the lower limit of normal (LLN, 0.00-0.75 mmol.L^-1^); upper limit of normal (ULN, 1.76 to 2.00 mmol.L^-1^) and four intermediate categories (0.75 to 1.00 mmol.L^-1^); (1.01 to 1.25 mmol.L^-1^); (1.26 to 1.50 mmol.L^-1^); (1.51 to 1.75 mmol.L^-1^).

To confirm that any association between Lac_TW _levels within the normal range and mortality was not being biased by patients who had individual lactate concentrations above 2 mmol.L^-1 ^while in the ICU, we then examined the association between Lac_TW _and mortality in the cohort of patients whose lactate never exceeded 2 mmol.L^-1^.

The primary outcome for analysis was hospital mortality and the secondary outcome was ICU mortality. We performed crude univariate analysis with lactate as a catagorial variable for comparison between groups according to hospital survival status using chi-square test for proportions, Student t-test for normally distributed outcomes and Wilcoxon rank sum tests otherwise. In addition, we performed multivariate analysis where we adjusted for all available predictors of hospital mortality included in the models (gender, age, APACHE II, mechanical ventilation, surgical admission and diagnosis type) determined by backward elimination of non-significant variables. Furthermore, to determine if the lactate associations were consistent across patient admission diagnosis subgroups and study hospitals, we examined the interactions between measures of lactate and other variables in the model. We report results from the multivariate models using odds ratios, OR (95% confidence intervals, 95% CI).

All analyses were performed using SAS version 9.2 (SAS Institute Inc, Cary, NC, USA). A two-sided *P*-value of 0.05 was considered to be statistically significant.

## Results

We studied a heterogeneous cohort of 7,155 critically ill patients with 172,723 blood lactate measurements (Table 1). The absolute blood lactate concentrations (admission lactate Lac_ADM_, maximal lactate Lac_MAX _and time-weighted lactate Lac_TW_), were significantly higher in non-survivors compared to survivors (Table 1).

**Table 1:** Clinical characteristics of hospital survivors and non-survivors

	n	Hospital Non-survivors	n	Hospital Survivors	*P*-value
**Male Sex**	1,561	**57.3% (894)**	5,590	**60.2% (3,365)**	**0.035**
**APACHE II score**	1,250	**24.6 (8.1)**	4,845	**15.1 (6.6)**	**< 0.0001**
**Age (yr)**	1,428	**65.8 (16.6)**	5,181	**59.7 (18.9)**	**< 0.0001**
**Number on mechanical ventilation**	1,434	**81.2% (1164)**	5,515	**55.6% (3,066)**	**< 0.0001**
**Surgical patients**	1,565	**28.4% (444)**	5,590	**48.7% (2,722)**	**< 0.0001**
**Diagnosis at admission**					
**Cardiac and vascular**	1,565	**26% (407)**	5,590	**21.6% (1,207)**	**0.0003**
**Thoracic and respiratory**	1,565	**18.5% (290)**	5,590	**18.9% (1,057)**	**0.69**
**Trauma**	1,565	**2.2% (34)**	5,590	**7.9% (442)**	**< 0.0001**
**Neurological**	1,565	**14.1% (221)**	5,590	**10.9% (609)**	**0.0004**
**Gastrointestinal tract diseases**	1,565	**14.1% (221)**	5,590	**22.9% (1,280)**	**< 0.0001**
**Other**	1,565	**25% (391)**	5,590	**17.7% (989)**	**< 0.0001**
**ICU stay (days)**	1,559	**3.0 (1.5 to 6.6)**	5,589	**2.5 (1.6 to 4.9)**	**< 0.0001**
**Hospital stay (days)**	1,312	**9 (3 to 24)**	5,131	**14 (8 to 29)**	**< 0.0001**
**Admission blood lactate** **(mmom.L^-1^)**	1,395	**2.3 (1.4 to 4.4)**	5,037	**1.5 (1.0 to 2.4)**	**< 0.0001**
**Time-weighted blood lactate (mmom.L^-1)^**	1,411	**2.0 (1.4 to 3.3)**	4,977	**1.3 (1.0 to 1.8**	**< 0.0001**
**Max blood lactate (mmom.L^-1)^**	1,565	**4.0 (2.2 to 7.5)**	5,590	**2.1 (1.4 to 3.3)**	**< 0.0001**

Data are expressed as, percentage (number), (standard deviation) or median (interquartile range).

APACHE II, Acute Physiology and Chronic Health Evaluation II.

### Overall assessment of hyperlactatemia (absolute hyperlactatemia)

**Figure 1:**
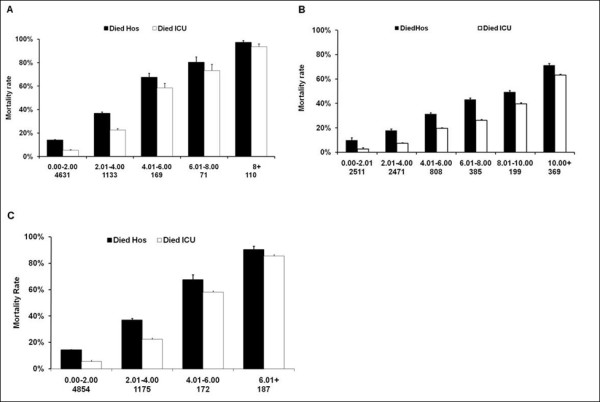
**Relationship among the admission, maximal and time weighted blood lactate concentration and mortality**. Relationship among the admission blood lactate concentration (Lac_ADM_) Panel **(a)**; maximal blood lactate concentration (Lac_MAX;_) Panel **(b)**; and time weighted blood lactate concentration (Lac_TW_) Panel **(c)**; and hospital and ICU mortality. The number of patients in each group is expressed as (n).

A higher crude Lac_ADM_, Lac_MAX _and Lac_TW _concentration above the reference range (0.00 to 2.00 mmol.L^-1^) was associated with a higher hospital and ICU mortality rate (Figure 1, Panel a, b, c, respectively). Multivariate analysis showed that compared to the current reference lactate concentration (0.00 to 2.00 mmol.L^-1^) a higher Lac_ADM _(> 8 mmol.L^-1^), Lac_MAX _(> 10 mmol.L^-1^) and Lac_TW _(> 6 mmol.L^-1^) blood lactate concentration was strongly associated with an increased adjusted hospital mortality (Lac_ADM _OR213.49 (95% CI 28.69 to 1588.71), *P *< 0.0001); Lac_MAX _OR8.44 (95% CI 5.99 to 11.91), *P *< 0.0001) Lac_TW _OR 37.78 (95% CI 18.72 to 76.25), *P *< 0.0001). This association between lactate (Lac_ADM_, Lac_MAX _and Lac_TW_) and adjusted mortality was independent of admission diagnosis, admission hospital and APACHE II score.

### Assessment of relative hyperlactatemia

**Figure 2:**
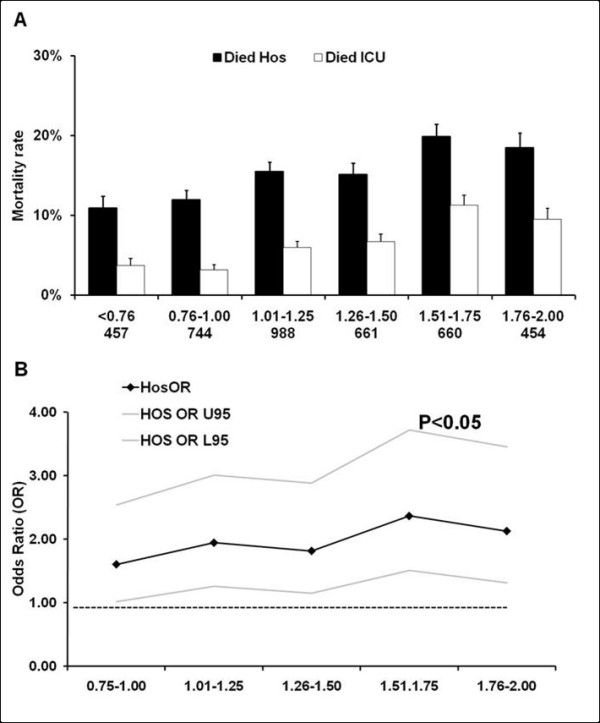
**Relationship between the admission blood lactate concentration within the *normal *range and mortality rate**. Relationship between the admission blood lactate (Lac_ADM_) concentration within the *normal *range and ICU and hospital mortality rate (Panel **(a)**). The number of patients in each group is expressed as (n). Panel **(b) **displays the result (adjusted odds ratios (OR) with 95% Confidence Interval (CI)) of a multivariate analysis assessing the association between admission blood lactate (Lac_ADM_) within the *normal *range and hospital mortality. (All ORs in the multivariate analysis are compared to the 0.00 to 0.75mmol.L^-1 ^group with the horizontal line representing an OR of 1.0.).

**Figure 3:**
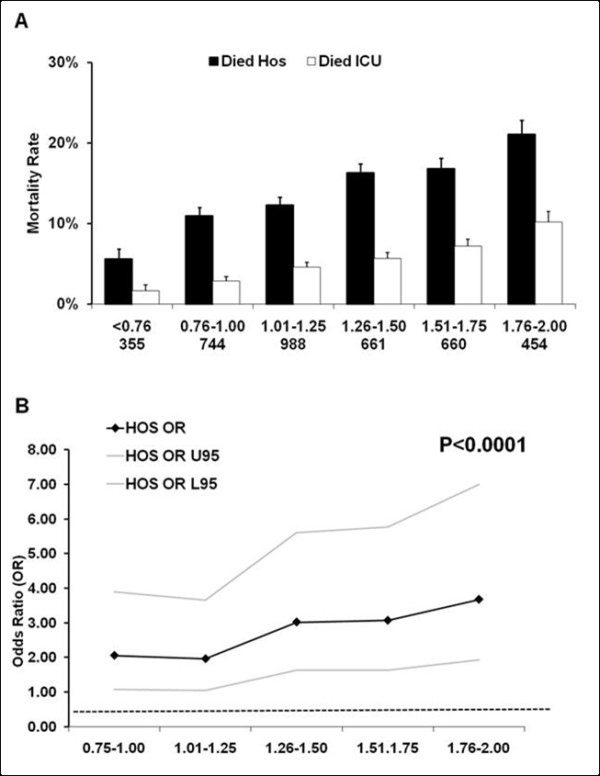
**Relationship between time-weighted blood lactate concentration within the *normal *range and mortality rate**. Relationship between time-weighted blood lactate (Lac_TW_) concentration within the reference range and ICU and hospital mortality rate (Panel **(a)**). The number of patients in each group is expressed as (n). Panel **(b) **displays the result (adjusted odds ratios (OR) with 95% Confidence Interval (CI)) of a multivariate analysis assessing the association between time-weighted blood lactate concentration (Lac_TW_) within the *normal *range and hospital mortality. Abbreviations: OR U95; odds ratio upper 95% CI; OR L95, odds ratios lower 95% CI. (All ORs in the multivariate analysis are compared to 0.00 to 0.75 mmol.L^-1 ^group with the horizontal line representing an OR of 1.0.).

We further identified the cohorts of patients with a Lac_ADM _(n = 3,964), Lac_MAX_, (n = 2,511) and with Lac_TW _(n = 4,584) within the current reference range (0.00 to 2.00 mmol.L^-1^). Table 2 shows the clinical characteristics of the Lac_ADM _subgroup of patients divided into hospital survivors and non-survivors. Patients with an admission or time weighted lactate level just below 2 mmol.L^-1 ^had a crude hospital mortality rate of approximately 20% (Figures 2a and 3b). Lac_ADM_, Lac_MAX _and Lac_TW _were significantly higher in hospital non-survivors compared to survivors (Table 2).

**Table 2:** Clinical characteristics for hospital survivors and non-survivors in patients with admission blood lactate concentration within the reference range

	n	Hospital Non-survivors	n	Hospital Survivors	*P*-value
**Male Sex**	607	**58.2% (353)**	3,357	**59% (1,981)**	**0.71**
**APACHE II score**	485	**22.1 (7.3)**	2,891	**14.2 (6.2)**	**< 0.0001**
**Age (yr)**	556	**66.0 (16.5)**	3,090	**60.7 (18.7)**	**< 0.0001**
**Number on mechanical ventilation**	551	**77.3% (426)**	3,313	**48.7% (1,613)**	**< 0.0001**
**Surgical patients**	607	**31% (188)**	3,357	**50.9% (1,709)**	**< 0.0001**
**Diagnosis at admission**					
**Cardiac and vascular**	607	**15.2% (92)**	3,357	**20.4% (685)**	**0.003**
**Thoracic and respiratory**	607	**24.2% (147)**	3,357	**20% (671)**	**0.017**
**Trauma**	607	**3.6% (22)**	3,357	**8.8% (295)**	**< 0.0001**
**Neurological**	607	**18.1% (110)**	3,357	**10.9% (366)**	**< 0.0001**
**Gastrointestinal tract diseases**	607	**15.3% (93)**	3,357	**23.7% (796)**	**< 0.0001**
**Other**	607	**23.6% (143)**	3,357	**16.2% (544)**	**< 0.0001**
**ICU stay (days)**	606	**3.0 (2.0 to 8.0)**	3,357	**2.0 (1.3 to 4.0)**	**< 0.0001**
**Hospital stay (days)**	501	**12.9 (5.2 to 30.2)**	3,064	**13.0 (7.9 to 26.7)**	**0.041**
**Admission blood lactate (mmom.L^-1^)**	607	**1.3 (1 to 1.6)**	3,357	**1.2 (0.9 to 1.5)**	**< 0.0001**
**Time-weighted blood lactate (mmom.L^-1^)**	599	**1.4 (1.1 to 1.9)**	3,212	**1.20 (1.0 to 1.5)**	**< 0.0001**
**Max blood lactate (mmom.L^-1^)**	607	**2.4 (1.6 to 4.1)**	3,357	**1.7 (1.3 to 2.4)**	**< 0.0001**

Data are expressed as, percentage (number), (standard deviation) or median (interquartile range) APACHE II, Acute Physiology and Chronic Health Evaluation II.

A higher admission lactate (Lac_ADM_) concentration within the reference range was associated with higher crude hospital mortality (Figure 2a), with a mortality rate of 18.5% in the higher risk cohort. There also was a significant independent relationship between Lac_ADM _within the reference range and adjusted hospital mortality (Figure 2b). Higher Lac_TW _within the reference range was independently associated with higher adjusted hospital mortality (Figure 3b) with a crude mortality rate of 21.1% in the higher risk cohort (Figure 3a). In addition, higher Lac_TW _(1.5 to 2.00 mmol.L^-1 ^vs 0.00 to 0.75 mmol.L^-1^) was also independently associated with hospital mortality in the cohort of patients whose lactate never exceeded 2 mmol.L^-1 ^(Lac_TW _OR4.8, 95% CI 1.8 to 12.4, *P *< 0.001, n = 2,254).

The association between adjusted hospital mortality and Lac_ADM _and Lac_TW _lactate concentrations within the normal range was first detected at lactate concentrations over 0.75 mmol.L^-1 ^and the strength of this association increased with higher lactate concentrations within the reference range (Figures 2b and 3b). The detected association between lactate within the reference range and adjusted hospital mortality was independent of admission diagnosis, admission hospital and APACHE II score. Interestingly, a higher crude and adjusted maximal lactate (Lac_MAX_) concentration within the *normal *reference range was not independently associated with increased hospital mortality (data not shown).

## Discussion

### Statement of key findings

We tested whether higher levels of lactatemia within the current reference range (relative hyperlactemia) are independently associated with an increased risk of hospital mortality. We found that most patients admitted to ICU had an admission or time weighted lactate level within the current normal reference range and yet a crude hospital mortality rate of approximately 20%. We also found that higher ICU admission (Lac_ADM_) and time weighted (Lac_TW_) blood lactate concentrations within the *normal *reference range were strongly and independently associated with hospital mortality. In addition, this increased mortality risk was first detected at lactate concentrations above 0.75 mmol.L^-1^.

### Comparison with previous studies

Many studies have found that either Lac_ADM _or Lac_MAX _above the reference range are associated with higher mortality following cardiothoracic surgery [12], trauma [7], major abdominal surgery [5], high risk surgery, major vascular trauma, sepsis [20], liver disease [21], in ventilated neonates [22] and critically ill children [11]. Our observations that the extent of absolute hyperlactatemia is strongly linked with mortality independent of admission diagnostic group in a large mixed cohort of critically ill patients confirm that lactate is a useful marker in the intensive care setting to identify patients at high risk of death. In addition, these findings suggest that other observations related to lactate obtained from our cohort might also be generalizable. In addition, we found that time weighted lactate (Lac_TW_), a representation of the lactate concentration throughout the ICU stay, was strongly associated with increasing hospital mortality. The finding that the duration of this derangement while in the ICU is associated with increased mortality expands previous work demonstrating that periods of sustained hyperlactataemia (that is, ongoing excess production or decreased clearance of lactate) is associated with an increased risk of death [3,5,23-28].

To our knowledge, this is the first study to assess the relationship of higher lactate concentrations within the current reference range and mortality. We found a strong association between an increased Lac_ADM _and Lac_TW _within the current reference range and increased hospital mortality. Furthermore, we demonstrated that higher Lac_TW _in the cohort of patients whose lactate ever exceeded 2 mmol.L^-1 ^was also strongly associated with higher hospital mortality. These results suggest that relative hyperlactaemia may be useful in identifying critically ill patients at high risk of death. Furthermore, we have demonstrated that the higher mortality associated with higher lactate levels (Lac_ADM _and Lac_TW_) within the normal reference range is detectable at all concentrations > 0.75 mmol.L^-1 ^compared to 0.00 to 0.75 mmol.L^-1^. In their aggregate, these results suggest that the transition from *physiological *to *pathological *lactatemia occurs at a concentration well below 2.00 mmol.L^-1 ^and that an elevated Lac_ADM _and/or Lac_TW _> 0.75 mmol.L^-1 ^identifies critically ill patients at higher risk of death.

### Implications for clinicians

These findings expand our understanding of lactate as a clinical biomarker in the ICU. Relatively small changes in lactate homeostasis as detected by higher blood concentrations within the reference range may reflect important otherwise undetected physiological changes, which may reflect widespread metabolic stress [29] and increased use of lactate as a fuel source [30].

A higher time weighted lactate (Lac_TW_) below 2 mmol.L^-1^, the cohort of patients whose lactate ever exceeded 2 mmol.L^-1^, was also strongly associated with increased hospital mortality. This finding extends our understanding of the reference range by emphasizing the role of the duration of lactate derangement in predicting increased risk of death [3,5,23-25,28,31,32]. Furthermore, it highlights the clinical importance of persistently higher lactate concentrations. This notion may explain why Lac_TW _(which reflects the extent and duration of the derangement) but not Lac_MAX _(which only reflects its momentary extent) predicted mortality within the reference range.

Our results suggest that ICU clinicians confronted with a patient with a Lac_ADM _or Lac_TW _(persistently higher lactate) over 0.75 mmol.L^-1 ^should look for any remediable causes of physiological stress and appreciate that these patients are at increased risk of an adverse outcome.

### Strengths and limitations of the study

The strengths of our study include the fact that it is the largest investigation of lactatemia in a general multicenter cohort of patients, thus carrying a higher degree of external validity. It used data from > 170,000 measurements obtained with state-of the-art technology, thus increasing their accuracy and reproducibility. It used robust and clinically relevant outcomes. It is the first to study the independent relationship between relative hyperlactatemia and outcome and identified clinically relevant findings. Limitations of the study include the fact that it is retrospective in design and thus potentially subject to systematic error and bias. However, all the clinical and electronic data utilised were collected prospectively in a large number of consecutive critically ill patients in four ICUs. The data are numerical in nature and were measured independently; thus they were not amenable to selection bias or unintended manipulation. A number of common ICU therapeutic interventions such as epinephrine [33], metformin [34], nucleoside analogues in HIV [35], high-volume hemofiltration (HVHF) with lactate-buffered replacement fluids [36] can all affect lactate levels and we did not have information on their use. We were therefore unable to include these in our multivariate analyses. However, the size of our study and the strength of the association between Lac_ADM _and Lac_TW _and mortality within the reference range independent of admission diagnosis and hospital suggest that these factors are not likely to have confounded the signal in this study. Despite this, clinicians should be aware of the potential of these iatrogenic causes of relative or absolute hyperlactataemia. Due to the smaller numbers of patients in the cohort with Lac_ADM _and Lac_TW _in the lowest three octiles, we compressed these octiles into a single group (0.00 to 0.75 mmol.L^-1^) to provide sufficient numbers for statistical analyses. This compression limited our ability to determine if higher blood lactate concentrations below 0.75 mmol.L^-1 ^may also be associated with increasing mortality.

### Future research

Our findings are novel and need to be confirmed by similar studies in other countries or patient populations before they can be considered to reflect a general biological principle. Such studies should ideally be performed prospectively with a simultaneous collection of information on interventions, which may affect lactate by dilution (intravenous fluids) or by changing its metabolism (drugs) and these studies should ideally also include non-ICU cohorts of patients (that is, Emergency Department patients). If these studies confirm the value of relative hyperlactatemia, the reference value for lactate in critically ill patients may require adjustment.

## Conclusions

In conclusion, higher Lac_ADM _and Lac_TW _blood lactate concentrations within the current reference range are associated with greater hospital mortality. These results suggest that even relative hyperlactaemia is a useful biomarker in critical illness. They also suggest that the upper level of the reference value for lactate in critically ill patients may require readjustment. Finally, they imply that clinicians should be especially alert in all patients with admission and/or persistent blood lactate concentrations within the current upper limit of the reference range.

## Key messages

• Blood lactate concentration is increasingly being measured in the critically ill.

• Higher intensive care unit blood lactate concentrations above the current *normal *range (absolute hyperlactatemia) are associated with increased hospital mortality.

• Higher intensive care unit admission blood lactate concentrations within the current *normal *range (relative hyperlactatemia) are associated with increased hospital mortality.

• Higher time weighted intensive care unit blood lactate concentrations within the current *normal *range (relative hyperlactatemia) are associated with increased hospital mortality.

• Higher blood lactate concentrations within the current normal range can be used to identify patients at high risk of death; possibly suggesting that we need to revise the current definition of normal blood lactate concentration in the critically ill.

## Abbreviations

ANZICS-APD: Australian and New Zealand Intensive Care Society - Adult Patient Database; ANZICS-CORE: Australian and New Zealand Intensive Care Society - Centre for Outcome and Resources Evaluation; APACHE: Acute Physiological and Chronic Health Evaluation; LLN: lower limit of normal; Lac_ADM_: admission lactate; Lac_MAX_: maximal lactate; Lac_TW_: time-weighted lactate; OR: odds ratio; ULN: upper limit of normal.

## Competing interests

The authors declare that they have no competing interests.

## Authors' contributions

AN, RB, VP, GH, D JC, MB and ES carried out the database searches, participated in the data collation and drafted the manuscript with AD, CF, ES, MR. AN, RB, VP, DJC, MB conceived of the study, and participated in its design and coordination and helped to draft the manuscript. All authors read and approved the final manuscript.
